# Effect of problem-based learning in the training of Central Sterile Supply Department nurses

**DOI:** 10.1371/journal.pone.0358870

**Published:** 2026-09-22

**Authors:** Sixin Jiang, Liangying Yi, Wei Pan, Hongxia Zhang, Xiaoxiao Liu, Caixia Yuan, Yaoguang Mou

**Affiliations:** 1 Department of Sterile Processing Nursing, West China Second University Hospital, Sichuan University, Chengdu, Sichuan, China; 2 Key Laboratory of Birth Defects and Related Diseases of Women and Children (Sichuan University), Ministry of Education, Chengdu, Sichuan, China; Johnson & Johnson, UNITED STATES OF AMERICA

## Abstract

**Background:**

Central Sterile Supply Department (CSSD) staff members’ mastery of professional knowledge and skills is associated with the quality of their work.

**Objectives:**

This study aimed to investigate the effect of problem-based learning in the training of CSSD nurses.

**Methods:**

This study was conducted from April to October 2024. Forty CSSD nurses at our hospital were randomly assigned to the observation group (n = 20) or the control group (n = 20) using a random number table. The observation group received problem-based learning, while the control group received traditional training methods. After six months of training, the two groups were compared in terms of theoretical knowledge, practical skills, self-directed learning competence, teamwork skills and satisfaction with training.

**Results:**

Scores for theoretical knowledge, practical skills, self-directed learning competence, teamwork skills and satisfaction with training were higher in the observation group than in the control group (*P* < 0.05).

**Conclusion:**

Problem-based learning could improve CSSD nurses’ professional knowledge, practical skills and satisfaction with training.

## Introduction

The Central Sterile Supply Department (CSSD) is responsible for cleaning, disinfecting and sterilizing reusable instruments, utensils and other items used in diagnosis and treatment, as well as supplying sterile items to other hospital departments [1,2]. The CSSD supports the normal operation of clinical diagnosis, treatment and nursing care, including the prevention and control of nosocomial infections [3,4]. The quality of CSSD work is directly associated with healthcare quality and patient safety. Therefore, the CSSD is a key department in the prevention and control of nosocomial infections [5].

CSSD staff in Australia, Germany and the United Kingdom can engage in CSSD work only after completing professional training and obtaining the relevant qualifications [6]. CSSD nurses in China graduate from nursing programs, but their education includes little or limited content on sterile supply. In line with the trend toward high-quality hospital development, increasing attention is being paid to the quality of CSSD work because of its distinctive characteristics and its role in preventing and controlling nosocomial infections. This has placed greater demands on the professionalism and standardized practices of CSSD nurses [7]. Therefore, on-the-job training is needed to improve CSSD nurses’ knowledge and skills in sterile supply. Such training can cultivate skilled professionals and ensure high-quality disinfection and sterilization services, thereby safeguarding clinical diagnosis, treatment and patient safety. Compared with other healthcare disciplines, sterile supply developed relatively late, and the training of CSSD nurses has lagged behind training for clinical nurses [8]. Therefore, improving CSSD nurse training is particularly important.

Problem-based learning is a problem-focused, learner-oriented and instructor-assisted training method [9]. It places learners in real problem situations and requires them to work through those situations. By consulting materials and collaborating to analyze and solve problems, learners can acquire knowledge, improve their problem-solving and self-directed learning skills and increase their enthusiasm for learning [10]. Group discussion is at the core of problem-based learning. Group members conduct research, discussion and study around a specific case. With participation and guidance from instructors, they solve problems that occur in clinical work through independent information gathering. This can improve learners’ independent learning and literature-search skills. Compared with traditional training methods, problem-based learning can stimulate learners’ enthusiasm, improve their hands-on skills and help them integrate theoretical knowledge with practical skills [11]. Problem-based learning also encourages learners to ask and answer questions.

Adult learners generally focus on the practical relevance of what they learn. They are concerned with whether the learning content is closely related to problems they encounter in their daily work. Applying problem-based learning to the training of CSSD nurses can increase their enthusiasm for learning despite the repetitive nature of their work, thereby helping to build a CSSD nursing team capable of meeting diverse healthcare demands. Problem-based learning has been widely applied in medical education. For example, Chen et al. combined case-based learning with problem-based learning in the ideological and political education component of the course Internal Medicine of Traditional Chinese Medicine [12]. Xiang applied problem-based learning to improve medical students’ ability to analyze complex cases in pathogenic biology [13]. Fan et al. reviewed recent developments in the research and application of problem-based learning in medical education [14]. However, studies on the application of problem-based learning in sterile supply remain scarce. This study applied problem-based learning to the on-the-job training of CSSD nurses to investigate its effectiveness and develop a training model that could be implemented regularly. The study was conducted among CSSD nurses at our hospital. The results showed that problem-based learning was more effective than the traditional training method.

## Methods

### Ethics approval and consent to participate

This study was conducted in accordance with the Declaration of Helsinki. All research methods were carried out in accordance with the relevant guidelines and regulations. This study was approved by the Medical Research Ethics Committee of West China Second University Hospital, Sichuan University [2024 Medical Scientific Research for Ethical Approval No. (050)]. Written informed consent was obtained from all participants prior to conducting the study.

### Study participants

This study was conducted among 40 CSSD nurses at our hospital from April to October 2024. A random number table was used to divide the nurses into two groups. The nurses who met all inclusion criteria and none of the exclusion criteria were numbered from 1 to 40 in chronological order according to their employment start dates. Forty unique numbers were selected from the random number table and matched one-to-one with the nurses. Nurses assigned odd numbers were allocated to the control group (n = 20), while those assigned even numbers were allocated to the observation group (n = 20). After grouping was completed, the lists of members in the two groups were retained by a CSSD quality controller who was not involved in the training or assessment conducted in this study. The instructors knew only the trainees in their respective groups. A single-blind evaluation was conducted during the assessment stage to reduce bias. The observation group received problem-based learning, while the control group received traditional training methods.

The inclusion criteria were as follows: (1) CSSD nurse who held a valid nursing practice certificate and (2) nurses who provided informed consent and voluntarily participated in the study. The exclusion criteria were as follows: (1) nursing interns, (2) rotating nurses, (3) visiting nurses and (4) nurses on leave for ≥ 6 months. The sample size was determined based on the feasibility of implementing the study. All CSSD nurses who met the inclusion criteria and none of the exclusion criteria were included, resulting in a total sample of 40 nurses.

Of the 20 nurses in the control group, two were male and 18 were female. Their mean age was 30.10 ± 3.58 years; four held an associate degree and 16 held a bachelor’s degree. Of the 20 nurses in the observation group, one was male and 19 were female. Their mean age was 31.00 ± 4.99 years; eight held an associate degree, 11 held a bachelor’s degree and one held a postgraduate qualification. No statistically significant differences were identified between the two groups in gender, age, professional title, educational attainment or years of work experience (*P* > 0.05), as shown in Table 1.

**Table 1:** Demographic information on CSSD nurses.

Items	Observation group n (%)	Control group n (%)	P
Gender			
Female	19 (95)	18 (90)	0.548
Male	1 (5)	2 (10)	
Age	31.00 ± 4.995	30.10 ± 3.582	0.517
Level of professional title			0.429
Junior	15 (75)	17 (85)	
Mid-level	5 (25)	3 (15)	
Educational attainment			0.196
Postgraduate qualification	1 (5)	0 (0)	
Bachelor’s degree	11 (55)	16 (80)	
Associate degree	8 (40)	4 (20)	
Years of work experience			0.752
≤ 5 years	9 (45)	10 (50)	
> 5 years	11 (55)	10 (50)	

## Research methodology

### Building a training team

The training team consisted of one researcher, two head nurses and two instructors. Each team member held a mid-level or senior professional title, had more than five years of work experience in the CSSD and had more than two years of training experience. The training team was divided into an instructional group and an assessment group. The instructional group provided training to the control group, developed cases for the observation group, implemented problem-based learning for the observation group and supervised the training process. The assessment group conducted assessments for both the control and observation groups. The assessors remained blind to group allocation throughout the assessments and independently assigned scores based solely on the nurses’ performance, thereby reducing subjective rating bias associated with group allocation.

### Developing the training plan

Following discussion, the training team developed a training plan based on the training goals, CSSD work requirements and CSSD nurses’ expectations of the training. The training plan covered basic theoretical knowledge, specialized knowledge and practical skills.

### Training implementation

Both the control and observation groups received six months of training with the same content, frequency and duration. The observation group participated in a case-based training session using problem-based learning every two weeks. Each case comprised two sessions, each lasting no more than one hour. Over the six-month period, the observation group completed six cases across 12 training sessions. Traditional training methods, including face-to-face lectures, practical skills demonstrations, instructional videos and live question-and-answer sessions, were used for the control group. Each training session lasted no more than 30 minutes, including five minutes for a live question-and-answer session. For practical skills, the nurses practiced independently after the demonstrations and instructional videos to become familiar with the procedures. Problem-based learning was implemented for the observation group according to the following steps:

### Preparation

Preparation was divided into four phases: (1) Instructor selection. In this study, members of the training team took turns serving as instructors according to their years of work experience. (2) Instructor training. The instructors completed the problem-based learning workshop provided by our hospital and attended demonstration classes on problem-based learning at the medical school. (3) Grouping and training for nurses. The nurses in the observation group were randomly divided into two subgroups, with 10 nurses in each subgroup. The training team provided the observation group with training on problem-based learning, including implementation methods, procedures, key considerations, classroom discussion requirements and assessment methods. (4) Training venue and equipment. A conference room was used as the training venue and was equipped with computers, internet access, projection equipment, a whiteboard and a conference table.

### Developing case materials

The training team developed case materials based on the training plan and problems encountered in practice, such as rust on instrument surfaces, failure to meet instrument-cleaning quality standards, errors in instrument assembly and packaging, failure to meet sterilization quality standards, abnormalities in sterilization monitoring and the occurrence of wet packs. During case development, the training team incorporated uncertain and challenging elements to stimulate the nurses’ critical thinking and motivation to solve problems. Given the characteristics of sterile supply knowledge, the cases were concise and not overly lengthy. Each case was studied over two sessions, with each session lasting no more than one hour.

### Problem-based learning

Problem-based learning was implemented in three phases: (1) Initial phase. The instructors sent case information to the two subgroups. The subgroup members studied the cases together, brainstormed and analyzed the problems and hypotheses based on their existing knowledge. If they could not solve a problem, they refined the unresolved problem to form a learning goal. The subgroup members recorded information for the problem-based learning discussion on the whiteboard, including known information about the cases, information to be consulted, a list of questions, identification and judgment and learning goals. In this phase, the instructors monitored each member’s participation in the case discussion, familiarized learners with the training model and demonstrated the thinking process through guidance and questioning. The learning goals were then assigned to individual members. The members conducted self-directed learning according to their learning goals, including but not limited to consulting the literature, reading expert consensus documents and consulting relevant criteria and standards from China and other countries. Relevant information was organized for study and sharing at the second session. Assigning learning goals was intended to improve learners’ sense of responsibility, increase their motivation to learn and give them opportunities to collaborate as a team. (2) Facilitation phase. At the beginning of the second training session, subgroup members shared the information they had gathered independently according to their assigned learning goals. They were expected to speak clearly, ask other members questions and discuss the issues. The instructors observed whether all subgroups members shared their independently gathered information and participated in the discussions. The instructors mediated any serious disagreements and encouraged members who did not express their opinions to contribute. This phase was intended to improve learners’ ability to express their views and formulate questions. The instructors then distributed materials for further study. Based on these materials, the learners determined whether the goals proposed in the previous step had helped solve the problems and whether the materials contained knowledge related to the learning goals. The learners then discussed whether any problems remained unresolved. If so, they proceeded to another learning cycle; if not, they moved to the next phase. (3) Ending phase. During the two training sessions for each case, the learners discussed and solved problems and summarized their knowledge through discussion and information sharing. They consolidated existing knowledge and acquired new knowledge through an iterative process of application, identification of knowledge gaps, learning, reapplication, identification of further knowledge gaps, relearning and communication to solve the problems.

## Outcome measures

### Assessments of theoretical knowledge and practical skills

Assessments of theoretical knowledge (basic knowledge, specialized knowledge, knowledge of nosocomial infections and knowledge of management) and practical skills (instrument cleaning, instrument packaging, loading for sterilization and biological monitoring) were conducted. Both assessments had a maximum score of 100. The assessment group of the training team conducted the assessments after completion of the training. The theoretical knowledge assessment took the form of a closed-book examination. The examination questions were drawn from the CSSD’s question bank. The question bank was developed by the training team according to the Central Sterile Supply Department (CSSD)-Part I: Management Standard (WS 310.1–2016), Central Sterile Supply Department (CSSD)-Part II: Standard for Operating Procedure of Cleaning, Disinfection and Sterilization (WS 310.2–2016) and Central Sterile Supply Department (CSSD)-Part III: Surveillance Standard for Cleaning, Disinfection and Sterilization (WS 310.3–2016), issued by the National Health and Family Planning Commission of China, as well as the Central Sterile Supply Department Training Manual (Version 2022) and the training goals. The questions were finalized after review and revision by two experts in sterile supply who held associate senior professional titles or higher. Reliability analysis showed that the Cronbach’s alpha coefficient of the question bank for the theoretical assessment was 0.911, indicating good internal consistency. The scoring method for practical skills was based on the Standard Operating Procedures for On-the-job Training in the Central Sterile Supply Department (Version 2022). The assessors were unaware of whether the nurses were assigned to the control or observation group (assessor blinding).

### Teamwork skills

The Clinical Teamwork Scale, developed by Guise et al. [15], was used to evaluate the CSSD nurses’ teamwork skills. The 12-item scale comprised six components: overall situation (maximum score: 10 points), communication (maximum score: 40 points), situational awareness (maximum score: 20 points), decision-making (maximum score: 10 points), role responsibility (maximum score: 30 points) and humanistic care (maximum score: 10 points). Each item was scored from 0 to 10 in ascending order. The maximum scale score was 120 points, with higher scores indicating stronger teamwork skills. The Cronbach’s alpha coefficient of the scale was 0.858.

### Self-directed learning competence

The self-directed learning competence scale, developed by Xiao et al. [16] in 2008, was used to evaluate the nurses’ self-directed learning competence. The Cronbach’s alpha coefficient of the scale was 0.944 and the content validity index was 0.970. The scale contained 34 items across four components: self-motivational beliefs (14 items), task analysis (6 items), self-monitoring and self-regulation (10 items) and self-evaluation (4 items). A 5-point Likert scale was used to rate the nurses’ responses. Response options were scored from 1 to 5 (“completely disagree,” “somewhat disagree,” “undecided,” “somewhat agree” and “completely agree”). Total scores ranged from 34 to 170, with higher scores indicating stronger self-directed learning competence. The Clinical Teamwork Scale and the self-directed learning competence scale have been published and have undergone reliability and validity testing. Both tools were used directly for assessment in this study.

### Satisfaction with training

An anonymous questionnaire was used to evaluate satisfaction with the different training methods. The satisfaction questionnaire was created using Wenjuanxing, a commonly used questionnaire survey platform in China. It contained the following eight items: (1) clarity of the training objectives; (2) focus of the training content; (3) instructor-learner interaction during training; (4) reasonableness of the training methods; (5) appropriateness of the training arrangements; (6) impact on increasing learners’ enthusiasm for learning; (7) impact on improving learners’ literature-search skills; and (8) impact on improving learners’ overall learning efficiency. A 5-point Likert scale was used to evaluate the nurses’ responses, ranging from 1 (“very dissatisfied”) to 5 (“very satisfied”). Higher scores indicated greater satisfaction (S1 Appendix). The questionnaire was designed by the researchers in this study and was reviewed and assessed by the training team and experts in sterile supply. Reliability analysis showed that the questionnaire’s Cronbach’s alpha coefficient was 0.825, indicating good internal consistency. The questionnaire was distributed and collected anonymously to reduce response bias.

### Statistical analysis

SPSS 25.0 was used for data analysis in this study. Normally distributed quantitative data were expressed as mean ± standard deviation ($\overset{―}{x}$±s) and compared using t-tests. Qualitative data were expressed as counts and percentages (%) and compared using the chi-square test. *P* < 0.05 was considered statistically significant (S1 Dataset).

## Results

### Assessments of theoretical knowledge and practical skills

Scores for theoretical knowledge and practical skills among CSSD nurses were significantly higher in the observation group than in the control group (*P* < 0.05), as shown in Table 2.

**Table 2:** Scores for theoretical knowledge and practical skills.

Groups	Theoretical knowledge				Score for theoretical knowledge	Score for practical skills
	Basic knowledge	Specialized knowledge	Knowledge of management	Knowledge of nosocomial infections		
Control group (n = 20)	19.10 ± 1.651	18.20 ± 2.041	18.65 ± 1.599	18.05 ± 1.959	74.00 ± 5.361	70.15 ± 5.234
Observation group (n = 20)	21.80 ± 1.765	22.50 ± 1.820	22.00 ± 1.806	22.95 ± 1.701	89.25 ± 3.370	82.30 ± 4.497
t	**–**4.996	**–**7.029	**–**6.211	**–**8.446	**–**10.771	**–**7.847
P	< 0.001	< 0.001	< 0.001	< 0.001	< 0.001	< 0.001

### Teamwork skills

Teamwork skill scores among CSSD nurses were significantly higher in the observation group than in the control group (*P* < 0.05), as shown in Table 3.

**Table 3:** Teamwork skill scores.

Groups	Overall situation	Situational awareness	Decision-making	Role responsibility	Humanistic care	Communication	Total
Control Group (n = 20)	7.15 ± 1.182	15.70 ± 1.525	7.95 ± 0.945	26.20 ± 1.704	8.15 ± 0.813	35.40 ± 1.875	100.55 ± 3.456
Observation group (n = 20)	8.10 ± 0.852	16.60 ± 1.095	8.60 ± 0.754	27.25 ± 1.209	8.10 ± 0.852	37.10 ± 1.373	105.75 ± 2.770
t	–2.915	–2.143	–2.405	–2.247	0.190	–3.272	–5.251
P	0.006	0.039	0.021	0.031	0.850	0.002	< 0.001

### Self-directed learning competence

Self-directed learning competence scores among CSSD nurses were significantly higher in the observation group than in the control group (*P* < 0.05), as shown in Table 4.

**Table 4:** Self-directed learning competence scores.

Groups	Self-motivational beliefs	Task analysis	Self-monitoring and self-regulation	Self-evaluation	Total
Control Group (n = 20)	39.55 ± 3.137	19.50 ± 2.800	35.45 ± 4.454	12.30 ± 2.105	106.80 ± 4.731
Observation group (n = 20)	55.55 ± 5.365	22.2 ± 2.353	38.70 ± 4.791	13.90 ± 1.651	130.35 ± 9.593
t	–11.513	–3.301	–2.222	–2.674	–9.846
P	< 0.001	0.002	0.032	0.011	< 0.001

### Satisfaction with training

Training satisfaction scores among CSSD nurses were significantly higher in the observation group than in the control group (*P* < 0.05), as shown in Table 5.

**Table 5:** Training satisfaction scores.

Items	Control group (n = 20)	Observation group (n = 20)	t	P
Clarity of the training objectives	4.80 ± 0.410	4.90 ± 0.308	–0.872	0.389
Focus of the training content	4.85 ± 0.366	4.95 ± 0.224	–1.042	0.304
Instructor-learner interaction during training	4.45 ± 0.510	4.80 ± 0.410	–2.390	0.022
Reasonableness of the training methods	4.35 ± 0.671	4.75 ± 0.550	–2.062	0.046
Appropriateness of the training arrangements	4.40 ± 0.681	4.85 ± 0.366	–2.604	0.013
Impact on increasing learners’ enthusiasm for learning	4.35 ± 0.587	4.75 ± 0.444	–2.430	0.020
Impact on improving learners’ literature-search skills	4.10 ± 0.788	4.90 ± 0.308	–4.229	< 0.001
Impact on improving learners’ overall learning efficiency	4.20 ± 0.523	4.65 ± 0.587	–2.559	0.015
Total	35.50 ± 2.306	38.55 ± 1.191	–5.256	< 0.001

## Discussion

### Problem-based learning could improve CSSD nurses’ theoretical knowledge and practical skills

The CSSD plays a vital role in preventing and controlling nosocomial infections, as well as in providing material support and improving the hospital’s ability to copy with public health emergencies [17,18]. CSSD nurse training in China generally relies on traditional methods that are not sufficiently targeted. These methods may neglect the development of nurses’ core competence and fail to increase their enthusiasm for learning and creativity [19,20]. Nurses who passively receive knowledge may have difficulty analyzing and solving problems. Continued dependence on instructors can lead to a lack of initiative and flexibility. In contrast, problem-based learning may encourage nurses to take greater initiative and actively acquire comprehensive, up-to-date knowledge in related fields from multiple sources. This may strengthen their ability to explore new information and retrieve relevant knowledge. Long-term use of this training method could help nurses identify effective ways to learn and master knowledge more efficiently.

### Problem-based learning could improve CSSD nurses’ teamwork skills

Working as a team in the CSSD is generally more efficient and less time-consuming than working individually. Problem-based learning centered on group discussion and allowed nurses to take active roles that differed from those in traditional training. In our study, members of each subgroup expressed their opinions on the same topic and communicated with each other. This helped expand and supplement the nurses’ knowledge and ideas, enabled them to develop solutions together and promoted collaborative learning, thereby improving their skills in organization, coordination and decision-making. It also helped nurses identify their own roles and responsibilities within the team. This improved their ability to use their knowledge and skills and helped them integrate more effectively into the CSSD team and contribute their expertise. The reporting, communication and question-and-answer sessions during training also helped nurses improve their communication and verbal expression skills when interacting with clinical staff.

### Problem-based learning could improve CSSD nurses’ self-directed learning competence

In our study, problem-based learning stimulated nurses to explore knowledge actively, helped them master basic knowledge through active learning and enabled them to apply their knowledge to practical tasks, improving their task analysis and problem-solving skills. In problem-based learning, nurses consulted the literature and analyzed and solved problems based on real cases. This could improve nurses’ self-directed learning competence, knowledge transfer skills and ability to innovate. The nurses gained more practical experience through repeated cycles of practice, reflection, further practice and further reflection, thereby improving their overall abilities. The roles of the instructors shifted from course instructors in traditional training to facilitators, guides and participants in problem-based learning, helping learners become independent and self-directed.

### Problem-based learning could improve CSSD nurses’ satisfaction with training

Problem-based learning is learner-centered, with the instructor acting as a facilitator. Our study used an interactive training method. The cases used for problem-based learning were based on real cases that occurred in the CSSD. This may have increased the nurses’ enthusiasm for learning. In problem-based learning, nurses independently consulted the literature, which could improve their literature-search skills and support their work and study. Therefore, CSSD nurses reported greater satisfaction with problem-based learning than with traditional training methods.

### Limitations

This study has several limitations. First, this was a single-center study with a small sample size (n = 40). Second, the nurses’ theoretical knowledge and practical skills were assessed by the assessment group of the training team. Although measures such as assessor blinding (the assessors were unaware of the nurses’ group assignments) and uniform scoring methods were implemented to control measurement bias, the fact that the same team carried out both training and assessment might have introduced potential bias. Third, this study evaluated the immediate effect of the training at the end of the training period but did not conduct a follow-up assessment of its long-term effects. The applicability and effects of problem-based learning in the CSSD require further validation. Future multicenter studies with larger sample sizes could be conducted. Such studies could use established assessment tools, independent blinded assessments and extended follow-up periods to further validate the effects of problem-based learning in CSSD nurse training and support its wider implementation.

## Conclusions

Problem-based learning has been widely applied in nursing training and has been shown to be effective. Our study showed that problem-based learning could improve CSSD nurses’ theoretical knowledge, practical skills, teamwork skills and self-directed learning competence and result in greater satisfaction among CSSD nurses. Our study provided a foundation for the professional development of CSSD personnel. Because this was a single-center study with a small sample size, the results may be applicable only to women’s and children’s hospitals. The results should therefore be generalized with caution.

## Supporting information

S1 Appendix Training satisfaction questionnaire for central sterile supply department nurses. (DOCX)

S1 Dataset Dataset of the study. (XLSX)
