# Supplementary material for: Effect of problem-based learning in the training of Central Sterile Supply Department nurses
Source: PLoS One. 2026 Sep 22;21(9):e0358870. doi: 10.1371/journal.pone.0358870 (PMC13596781; doi:10.1371/journal.pone.0358870)
Supplement: Supplementary file 1 [file pone.0358870.s001.docx]

**S1 Appendix**

**Training satisfaction questionnaire for Central Sterile Supply Department nurses**

**Instructions**

1. This is an anonymous questionnaire survey intended for training research purposes. Your responses will not be used to assess your learning outcomes or work performance. All data are strictly confidential. Please answer truthfully and objectively based on your own experiences.
2. The questionnaire contains one basic information question, eight rating questions, and one open-ended question. For the rating questions, a 5-point Likert scale is used to measure your satisfaction with CSSD training: 1 = very dissatisfied, 2 = dissatisfied, 3 = neutral, 4 = satisfied, and 5 = very satisfied.
3. Please mark “√” in the corresponding box for each question in Parts I and II. Only one response option should be selected for each question. Do not leave any questions unanswered. The questionnaire will take approximately 3 minutes to complete.

**Part I. Basic information**

1. What type of training did you receive?

□ Problem-based learning　　□ Traditional training methods

**Part II. Training satisfaction rating**

Please rate your satisfaction with each of the following items:

| No. | Items | Very dissatisfied (1 point) | Dissatisfied (2 points) | Neutral (3 points) | Satisfied (4 points) | Very satisfied (5 points) |
| --- | --- | --- | --- | --- | --- | --- |
| 1 | Clarity of the training objectives | □ | □ | □ | □ | □ |
| 2 | Focus of the training content | □ | □ | □ | □ | □ |
| 3 | Instructor-learner interaction during training | □ | □ | □ | □ | □ |
| 4 | Reasonableness of the training methods | □ | □ | □ | □ | □ |
| 5 | Appropriateness of the training arrangements | □ | □ | □ | □ | □ |
| 6 | Impact on increasing your enthusiasm for learning | □ | □ | □ | □ | □ |
| 7 | Impact on improving your literature-search skills | □ | □ | □ | □ | □ |
| 8 | Impact on improving your overall learning efficiency | □ | □ | □ | □ | □ |

**Part III. Opinions and suggestions**

What areas of this training need improvement? (Optional)

_________________________________________________________________________________________________________________________________________________________________________________________________________________________________

Thank you sincerely for taking the time to participate in this survey.
